# IL-1 and TNF mediates IL-6 signaling at the maternal-fetal interface during intrauterine inflammation

**DOI:** 10.3389/fimmu.2024.1416162

**Published:** 2024-06-04

**Authors:** Pietro Presicce, Cynthia Roland, Paranthaman Senthamaraikannan, Monica Cappelletti, McKensie Hammons, Lisa A. Miller, Alan H. Jobe, Claire A. Chougnet, Emily DeFranco, Suhas G. Kallapur

**Affiliations:** ^1^ Divisions of Neonatology and Developmental Biology, David Geffen School of Medicine at the University of California, Los Angeles, Los Angeles, CA, United States; ^2^ Department of Obstetrics/Gynecology, Maternal-Fetal Medicine, University of Cincinnati, Cincinnati, OH, United States; ^3^ Division of Neonatology/Pulmonary Biology, Cincinnati Children’s Hospital Research Foundation, and the University of Cincinnati College of Medicine, Cincinnati, OH, United States; ^4^ Division of Immunogenetics, David Geffen School of Medicine at the University of California, Los Angeles, Los Angeles, CA, United States; ^5^ Department of Anatomy, Physiology, and Cell Biology, School of Veterinary Medicine, University of California, Davis, Davis, CA, United States; ^6^ Immunobiology, Cincinnati Children’s Hospital Research Foundation, and the University of Cincinnati College of Medicine, Cincinnati, OH, United States

**Keywords:** chorioamnionitis, amnion, inflammation, innate immunity, reproductive immunology

## Abstract

**Introduction:**

IL6 signaling plays an important role in triggering labor and IL6 is an established biomarker of intrauterine infection/inflammation (IUI) driven preterm labor (PTL). The biology of IL6 during IUI at the maternal-fetal interface was investigated in samples from human subjects and non-human primates (NHP).

**Methods:**

Pregnant women with histologic chorioamnionitis diagnosed by placenta histology were recruited (n=28 term, n=43 for preterm pregnancies from 26-36 completed weeks of gestation). IUI was induced in Rhesus macaque by intraamniotic injection of lipopolysachharide (LPS, n=23). IL1 signaling was blocked using Anakinra (human IL-1 receptor antagonist, n=13), and Tumor necrosis factor (TNF) signaling was blocked by anti TNF-antibody (Adalimumab n=14). The blockers were given before LPS. All animals including controls (intraamniotic injection of saline n=27), were delivered 16h after LPS/saline exposure at about 80% gestation.

**Results:**

IUI induced a robust expression of *IL6* mRNAs in the fetal membranes (chorion-amnion-decidua tissue) both in humans (term and preterm) and NHP. The major sources of *IL6* mRNA expression were the amnion mesenchymal cells (AMC) and decidua stroma cells. Additionally, during IUI in the NHP, *ADAM17* (a protease that cleaves membrane bound IL6 receptor (IL6R) to release a soluble form) and *IL6R* mRNA increased in the fetal membranes, and the ratio of IL6 and soluble forms of IL6R, gp130 increased in the amniotic fluid signifying upregulation of IL6 trans-signaling. Both IL1 and TNF blockade suppressed LPS-induced *IL6* mRNAs in the AMC and variably decreased elements of IL6 trans-signaling.

**Discussion:**

These data suggest that IL1 and TNF blockers may be useful anti-inflammatory agents via suppression of IL6 signaling at the maternal-fetal interface.

## Introduction

Intrauterine infection or inflammation (IUI) is a major cause of preterm labor/preterm births (PTL/PTB) (1). In addition to causing prematurity, IUI also causes fetal inflammation with injury to fetal lung, gastrointestinal tract and brain (2–4), further compounding morbidity in the preterm neonate. To date, the most validated marker of IUI is interleukin 6 (IL6) levels in the amniotic fluid (AF), cervical fluid, and cord blood (5–10). IL6 also appears to be involved in the inflammatory cascade leading to PTL because clinical studies show a dose response of AF IL6 with the incidence of PTL, and inhibition of IL6 signaling decreased IUI induced PTB in mice (11–13). We recently reported that in IUI models of Rhesus macaques, a higher expression of IL6 signaling pathways in the fetal membranes is needed to induce PTL (14). Thus, clinical and experimental data strongly implicate IL6 signaling pathway in the causation of PTL/PTB.

IL6 is a pleitropic cytokine expressed by multiple cell types (15), and is known to have both pro- and anti-inflammatory signaling properties in multiple tissues (16). IL6 signaling occurs via three distinct mechanisms. Classic signaling requires a membrane-bound IL6 receptor which forms a complex with the ubiqutoiusly expressed gp130 protein. This complex then activates the JAK-STAT pathway, particularly STAT3. This classic signaling is thought to be regenerative, homeostatic, or anti-inflammatory in nature. In contrast, IL6 trans-signaling occurs when IL6 binds to a soluble IL6 receptor (sIL6R) which then allows IL6 signaling via gp130 in cells not expressing the membrane-bound IL6R and is pro-inflammatory (15, 17). A recently identified third form of signaling called cluster signaling involves preformed complexes of membrane-bound IL6–mIL6R on one cell activating gp130 subunits on target cells (18). Soluble glycoprotein130 (sgp130) by binding to circulating IL6 inhibits IL6 trans-signaling and sgp130 levels decrease in pregnancies complicated by IUI (19, 20). Thus, the ratios of IL6/sIL6R and IL6/sgp130 have often been used as an indicator of IL6 trans-signaling (20–23).

IL1 and TNF are potent cytokines that regulate and orchestrate multiple aspects of immune response. IL1 expression is regulated very tightly with both transcriptional and translational level of control involving the inflammasome complex, which regulates caspase-1 that ultimately cleaves pro-IL1β to active IL1β (24). In clinical trials for cardio-vascular diseases, IL1 inhibitors decrease IL6 signaling (25). We reported that in a preterm Rhesus macaque model of IUI induced by intraamniotic injection of LPS neutrophil infiltration of the fetal membranes, neutrophil activation, and pro-inflammatory mediators in the intrauterine compartments are significantly decreased by inhibition of IL1 or TNF signaling (26, 27). We therefore hypothesized that IL6 signaling during IUI is regulated by IL1 and TNF signaling. IL6 signaling in fetal membranes was compared in women with IUI delivering at preterm and term gestation vs. gestation matched no IUI subjects. Next, we inhibited IL1 or TNF signaling in the Rhesus macaques IUI model with recombinant human IL-1 receptor antagonist (Anakinra) or Adalimumab (anti-TNF antibody). We focused on the fetal membranes as a target tissue for evaluation of IL6 signaling and cell type expression of IL6 since this tissue has the most differential gene expression during IUI (28).

## Materials and methods

### Human samples

Twenty-eight pregnant women with term pregnancies (>37 weeks of gestation) and forty-three pregnant women with preterm pregnancies from 26^0^ to 36^6^ weeks of gestation were recruited. Maternal and neonatal demographic characteristics of the cohorts are shown in **Supplementary Table 1**. IUI was diagnosed by placenta pathology of histologic chorioamnionitis (Chorio) based on Redline’s criteria (29). Cohorts were developed based on Chorio positive term and preterm samples. Some preterm samples were used in previous studies (26). The numbers of samples for each experiment are shown in the corresponding figure.

### Animals

Time-mated female Rhesus macaques received 1 ml saline solution (n=27) or 1 mg LPS (n=23, MilliporeSigma) in 1 ml saline solution by ultrasound guided intra-amniotic (IA) injection. LPS induced inflammation was blocked by rhIL-1RA (n=13; Anakinra, Sobi) given to the pregnant monkey intra-amntiotically (IA) (50 mg) + maternal subcutaneous (SC) (100 mg) 1 and 3 hours before LPS respectively, or Tumor necrosis factor (TNF) blocker Adalimumab (n=14; Humira, AbbVie Inc. North Chicago, IL) given IA (40 mg) + SC (40 mg) 1 and 3 h before LPS respectively, as previously described (26, 27, 30). Dams were surgically delivered 16 hours later since our previous results demonstrate that most inflammatory markers were higher at 16 hours compared with longer exposures (**Supplementary Figure 1**) (14, 27, 30). Some animals were used in previous studies (26, 27, 30, 31). Maternal and neonatal demographic characteristics of the cohorts are shown in **Supplementary Table 2**. There were no spontaneous deaths or preterm labor in the animals. It was not always possible to obtain all the tissues/fluids from each animal. The numbers of animals for each experiment are shown in the corresponding figure. Sample integrity during varying periods of freezer preservation for different samples was verified and assays with all samples for a particular experiment were run at the same time.

### Extra-placental membranes isolation

Human and Rhesus extra-placental chorioamnion-decidua (CAMD) fetal membranes were collected within 30 minutes of delivery, dissected away from the placenta, and prepared as previously described (26, 27, 32). To study the contribution of each layer within the fetal membranes, amnion, chorion, and decidua parietalis were physically separated within 30 minutes of delivery and snap-frozen for RNA studies.

### *In situ* RNA hybridization and immunofluorescence

*In situ* RNA hybridization and Immunofluorescence dual staining was performed using RNAscope technology (Advanced Cell Diagnostics, Hayward, California) following the manufacturer’s protocol. Briefly, formalin fixed paraffin embedded Rhesus and human chorioamnion-decidua (CAMD) was cut into 5 μm sections and mounted on Poly L-Lysine adhesive coated glass slides (Newcomersupply, Middleton, WI). After de-paraffinization for 1h at 60°C, slides were treated for 10 min with Pretreatment 1 solution at room temperature. Subsequently the slides were treated at boiling with Pretreatment 2 solution for 15 min, followed by Pretreatment 3 (protease) for 30 min at 40°C. Following pretreatments, the sections were hybridized with IL-6 probes (RNA scope LS2.5 probe - MMS-497321 and HS-310371, ACD) for 2 hours at 42°C followed by RNA scope amplification (RNAscope 2.5 HD reagent kit, cat.# 322350) and FAST RED chromogenic substrate was used for visualization of staining. As positive and negative controls, RNAscope 2.5 probe-Ppib and RNAscope 2.5 probe_dapB were used. After *In situ* hybridization step, the slides were subjected to immunofluorescence staining to co-localize different proteins by incubation with either anti-human vimentin (Cat.# SC7557; dilution 1:200, Santa Cruz), Pancytokeratin (Cat.#SC81714; dilution 1:100, Santa Cruz), CD45 (CD45–2B11; Cat# 14–9457-82; dilution 1:50, Thermofisher) in 10% normal horse serum/0.2% Tween 20 at 4°C overnight. Staining was visualized using fluorescently labeled secondary antibodies (AF488; 1:200 dilution; Invitrogen) for 1 hour at room temperature. Nuclear counterstain was achieved using ProLong Gold antifade with DAPI. Stained slides were imaged on Leica microscopy. Images were collected using a Zeiss Axioplan 2 microscope and AxioVision 4 software (Zeiss).

### IL6, soluble IL6 receptor (sIL6R) and soluble gp130 (sgp130) ELISA

sIL-6R and sgp130 levels in rhesus amniotic fluid were determined by Rhesus macaque IL-6R ELISA kit (cat.# - ELK-IL6sR; RayBio, Norcross, GA) and Human Soluble gp130 Quantikine ELISA kit (cat.# - DGP00; R&D Systems, Minneapolis, MN) following manufacturer’s instructions.

### RNA Isolation, cDNA generation and quantitative RT-PCR

Total RNA was extracted from snap frozen rhesus tissues after homogenizing in TRIzol. RNA concentration and quality was measured by Nanodrop Spectrophotometer. Reverse transcription of RNA was performed using Verso cDNA synthesis kit (Thermo Fisher Scientific, Grand Island, NY) following manufacturer’s protocol. Quantitative RT-PCR (qPCR) was carried out in a StepOnePlus Real Time PCR system (Applied Biosystems) following standard cycling conditions. qPCR assays were performed with Rhesus- and human-specific TaqMan gene expression primers (Thermo-Fisher Scientific, Grand Island, NY). The Eukaryotic 18S mRNA was an endogenous control for normalization of the target RNAs and a sample from an IA saline animal or human preterm chorio negative sample was used as a calibrator. The values were expressed relative to the average value of the control group. For quantification, average of 5 randomly selected HPF fields were plotted as the representative value for the sample. Counts were performed in a blinded manner.

### Statistical analyses

GraphPad Prism software (version 9.0) was used to graph and analyze statistical significance. Values were expressed as means ± SEM. Two-tailed Mann-Whitney *U* tests (for non-normally distributed continuous variables), 2-tailed Student *t* test (for Gaussian distributed data points), and Fisher’s exact test (for categorical variables) were used to determine differences between groups. Results were considered significant for *P* ≤ 0.05.

## Results

### IUI induced *IL6* gene expression in term and preterm choarioamnion-decidua samples from human subjects

**Figure 1:**
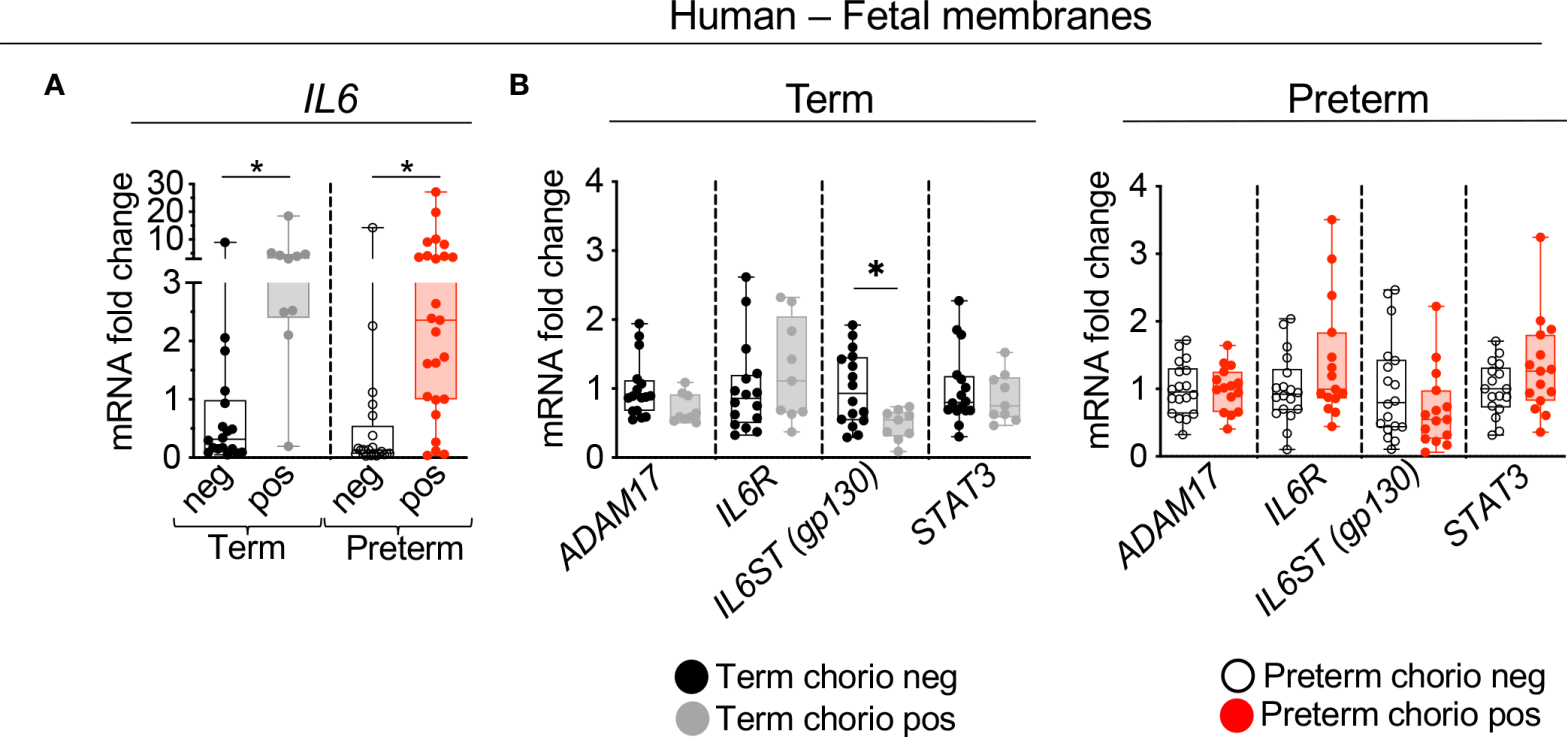
Chorioamnionitis increased the expression of *IL6* mRNA in human term and preterm fetal membranes. Human extraplacental chorioamnion-decidua (CAMD, fetal membranes) samples from women that delivered at term or preterm were obtained within 30 minutes of delivery and chorioamnionitis was diagnosed by placenta histology. **(A)** Chorioamnionitis (chorio) induces a significant increase of *IL6* mRNA expression in both preterm and term samples. **(B)** Chorio induces a significant decrease of *IL6ST* (*gp130*) in term CAMD samples compared to term without chorio (Term chorio neg) samples. (Term chorio neg n=18; Term chorio pos n=9–10; Preterm chorio neg n=18–21; Preterm chorio pos n=15–25). Expression of gene mRNA was measured by quantitative PCR (Taqman probes) and average mRNA values are fold increases over the average value for no chorio after internally normalizing to the housekeeping 18S RNA. Data are mean ± SEM, *p < 0.05 vs. samples without chorio (Mann–Whitney U-test).

**Figure 2:**
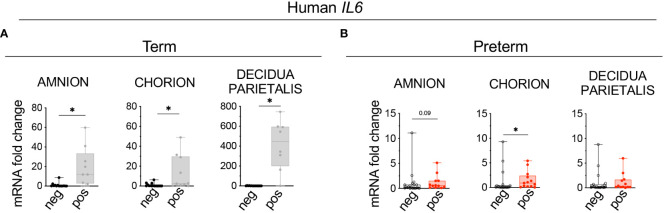
Chorio increased significantly the expression of *IL6* mRNA in each of the three tissues (amnion, chorion, decidua) of term samples and in the chorion of preterm samples. Amnion, chorion, and decidua parietalis were physically separated. **(A)** Chorio induces a significant increase of *IL6* mRNA expression in the three layers of term samples (Term neg n=15; Term pos n=10). **(B)** Similar results were observed in the chorion of preterm samples, (Preterm chorio neg n=18–19; Preterm chorio pos n=12–13). Data are mean ± SEM, *p < 0.05 vs. samples without chorio (Mann–Whitney U-test).

Expression of genes in the IL6 signaling pathway were assessed in our IUI cohort in both term and preterm gestation subjects (see **Supplementary Table 1** for demographic and clinical details). First, the expression levels were assessed in the extraplacental fetal membranes comprising the amnion, chorion, and decidua parietalis. Consistent with previous reports, *IL6* expression increased ~2.5 fold in both term and preterm samples (**Figure 1A**). The expression of *ADAM17* (encoding a protease that releases membrane bound IL6 receptor to a soluble form (sIL6R)), *IL6R*, and the downstream transcription factor *STAT3* did not change after IUI (**Figure 1B**). In the term but not preterm samples, there was a decrease in *IL6ST* (*gp130*) expression (**Figure 1B**). Next, the three different layers of fetal membranes were physically separated to understand the tissue specificity of induction of IL6 pathway. In term samples, IUI induced a significant increase of *IL6* mRNA in all the three layers compared to no IUI samples (**Figure 2A**). In the preterm compared to no IUI samples, there was a trend toward increased expression of *IL6* in the amnion (p=0.09; **Figure 2B**). In preterm chorion but not decidua samples, IUI induced an increase in *IL6* expression (**Figure 2B**), Of note, term samples show a higher magnitude of fold increase compared to preterm samples (compare **Figures 2A, B**).

### Decidua stromal cells and amniotic mesenchymal cells are the major source of *IL-6* during IUI

**Figure 3:**
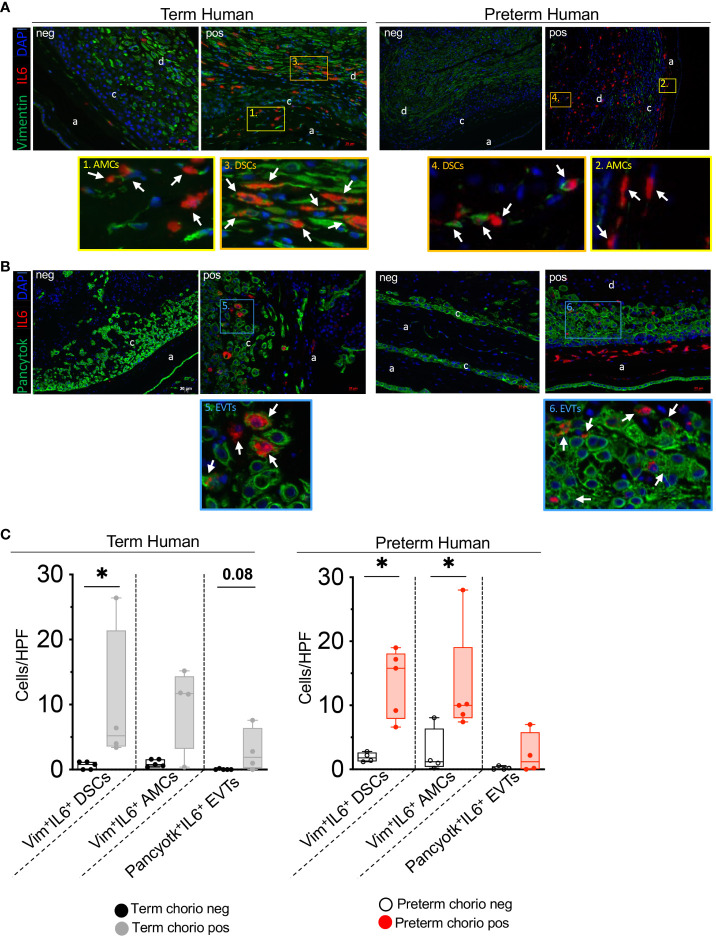
Chorio significantly increased the number of human *IL6* ^+^ stroma cells in the decidua parietalis and *IL6* ^+^ mesenchymal cells in the amnion. Fixed fetal membranes (chorion-amnion-decidua parietalis) paraffin embedded sections were stained by immuno-colocalization. **(A)** Representative images showing *IL6* mRNA identified by RNAscope *in situ* hybridization and Vimentin (Vim) colocalization by immunofluorescence. *IL6* is shown in red and Vim in green. White arrows in the magnified yellow inset (#1-term, #2-preterm) indicate Vim^+^ *IL6* ^+^ Amniotic Mesenchymal Cells (AMCs), while white arrows in the magnified orange inset (#3-term, #4-preterm) indicate Vim^+^ *IL6* ^+^ Decidual Stromal Cells (DSCs). **(B)** Representative images showing *IL6* mRNA identified by RNAscope *in situ* hybridization and Pan-cytokeratin (Pancytok) colocalization by immunofluorescence. *IL6* is shown in red and Pancytok in green. DAPI indicates nuclear staining (blue) in all images. White arrows in the magnified blue inset indicate Pancytok^+^ *IL6* ^+^ Extra Villous Trophoblast (EVTs) in both Term pos (inset #5) and Preterm pos (inset #6) subjects. (a, amnion; c, chorion, and d, decidua) **(C)** Quantification of Vim^+^ DSC, AMC, and Pancytok^+^ EVT cells expressing *IL6* in amnion, chorion, and decidua. Average of 5 randomly selected HPF fields were plotted as the representative value for the sample. Counts were performed in a blinded manner. Data are mean ± SEM. HPF, high-power field; (Term neg n=5; Term pos n=4), (Preterm chorio neg n=4; Preterm chorio pos n=5). *p < 0.05 (Mann–Whitney U-test).

To understand which cells express *IL6*, immuno-colocalization studies were done using established markers Vimentin (mesenchymal cells) and Pancytokeratin (trophoblastic cells) (33, 34). In term subjects, *IL6* expression increased significantly in the decidua stroma cells (DSC) with trends in amnion mesenchymal cells (AMC) and extra villous trophoblast (EVT) in the chorion (**Figures 3A, C**). In the preterm subjects both DSC and AMC expression of *IL6* increased significantly with trend toward an increase in the EVT (**Figures 3B, C**). In the term population DSC was the major cell type expressing *IL6*, while in the preterm both DSC and AMC were dominant cell types expressing *IL6*. To understand contributions from immune cells, *IL6* mRNA detection was combined with immune-colocalization with CD45. Regardless of the presence of inflammation, in both term and preterm human fetal membranes, there were very few CD45^+^ immune cells expressing *IL6*, and the majority of the cells expressing *IL6* were the non-immune CD45^-^ cells (**Supplementary Figures 2A, B**). Similar results were observed in the Rhesus macaque (**Supplementary Figures 3A, B**).

### IL-1ra and TNF-blockade decrease the expression of components of IL6 signaling pathway in Rhesus IUI induced by LPS

**Figure 4:**
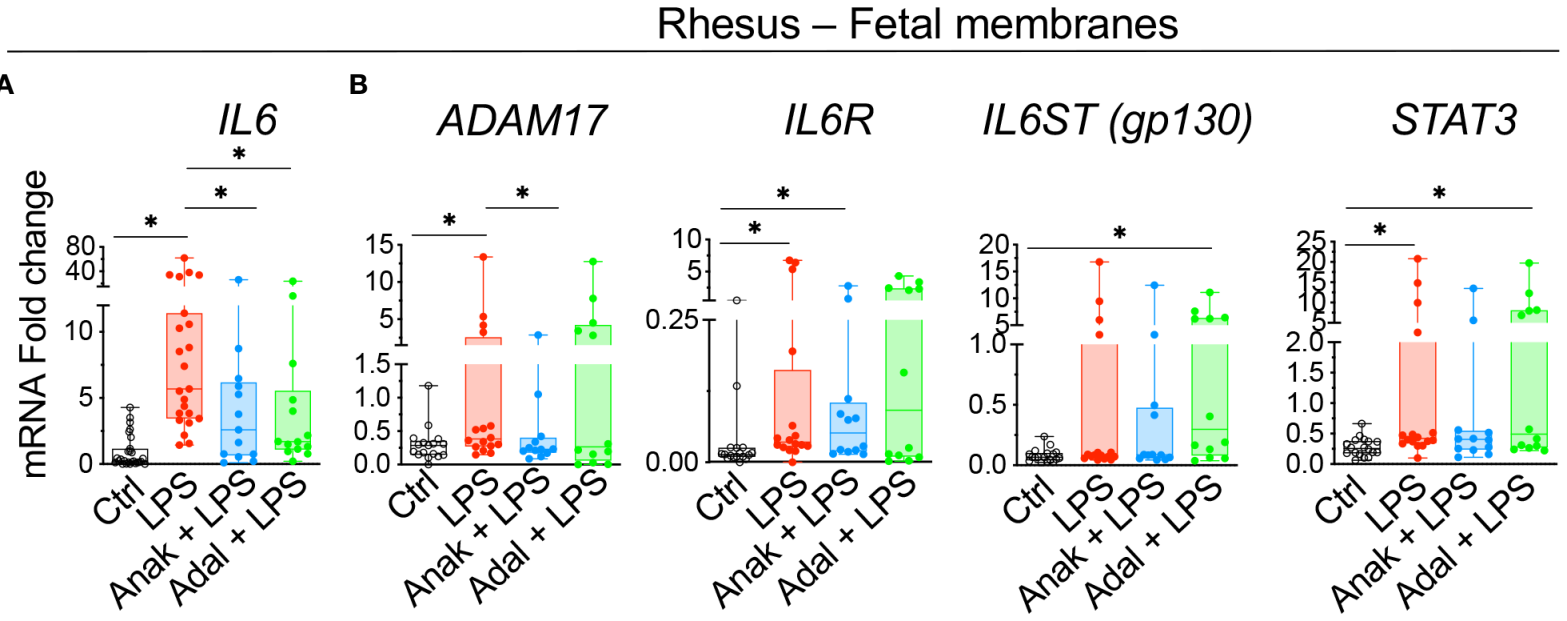
Chorioamnionitis increased the expression of *IL6* mRNA in rhesus preterm fetal membranes in a IL1- and TNF-dependent fashion. Chorioamnionitis was induced by intraamniotic (IA) injection of LPS. Controls (Ctrl) received intraamniotic saline. Rhesus extraplacental chorioamnion-decidua (fetal membranes) samples were obtained at delivery 16h after IA LPS/saline and chorioamnionitis (chorio) was diagnosed by placenta histology. mRNAs for molecules in *IL6* signaling were measured. **(A)** Chorio induces a significant increase of *IL6* mRNA expression. Both inhibitors Anakinra (Anak) and Adalimumab (Adal) significantly decreased *IL6* mRNA expression compared to LPS-exposed animals. (Ctrl n=27; LPS n=23; Anak+LPS n=13; Adal+LPS n=14). **(B)** Chorio induces a significant increase of *ADAM17*, *IL6R*, and *STAT3* mRNAs in LPS-exposed samples compared to ctrl samples. Anakinra but not Adalimumab decreased LPS-induced *ADAM17* mRNA. (Ctrl n=18; LPS n=16; Anak+LPS n=12; Adal+LPS n=12). Average mRNA values are fold increases over the average value for no chorio (dotted line) after internally normalizing to the housekeeping 18S RNA. Data are mean ± SEM, *p < 0.05 (Mann–Whitney U-test).

To understand mechanisms of IL6 regulation, experiments were done in our previously established preterm Rhesus macaque model of IUI induced by intraamniotic injection of LPS, where the timing of IUI is known and efficacy of the inhibitors was established (26, 27). Similar to human subjects, *IL6* expression increased in the Rhesus chorion-amnion-decidua parietalis tissue compared to saline controls (**Figure 4A**). Unlike the humans, *ADAM17*, *IL6R* and *STAT3* expression increased after LPS exposure (**Figure 4B**). There was no change in *IL6ST* (*gp130*) expression. Both IL1 and TNF inhibition decreased *IL6* mRNA expression in the fetal membranes (**Figure 4A**). IL1 but not TNF inhibition decreased *ADAM17* expression, but neither inhibitors significantly reduced *IL6R* and *STAT3* expression (**Figure 4B**).

**Figure 5:**
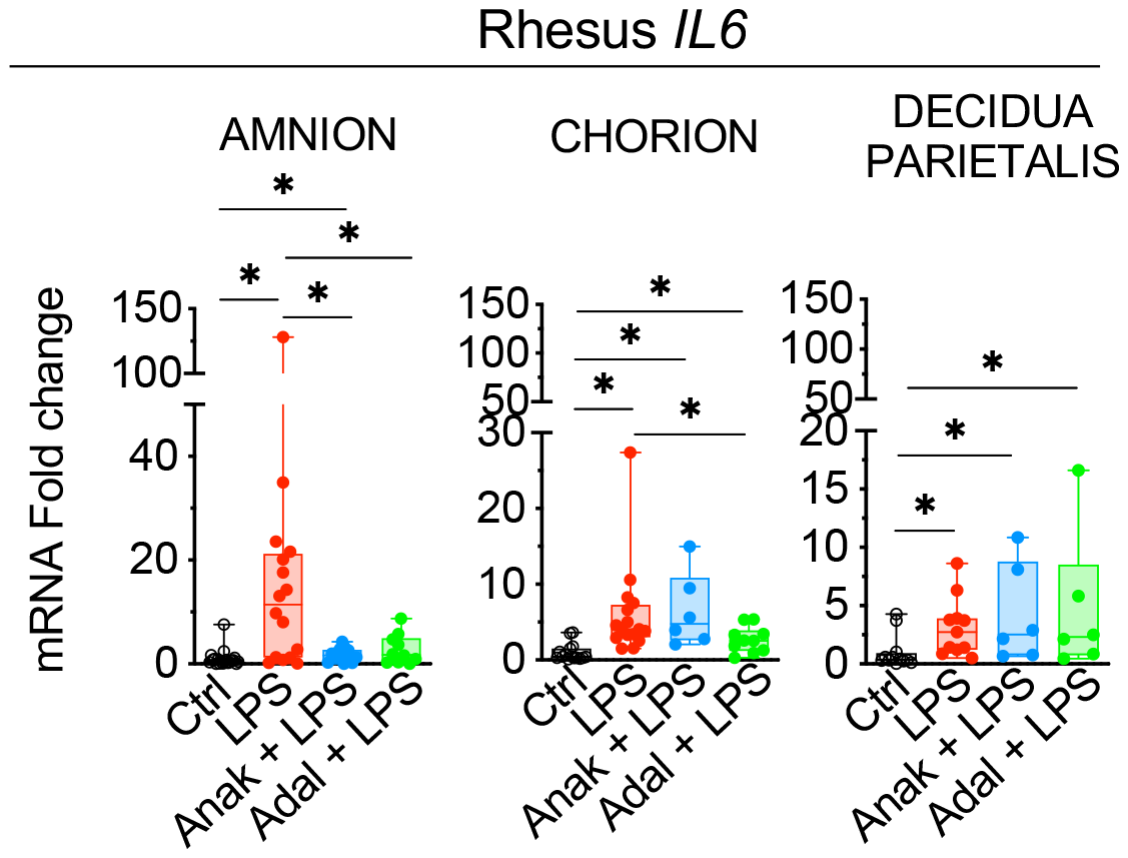
Partial decrease of LPS-induced *IL6* mRNAs in fetal membranes by Anakinra and Adalimumab. Amnion, chorion, and decidua parietalis were physically separated. LPS-exposure induced a significant increase of *IL6* mRNA expression in the three tissue layers. Anakinra decreased LPS-induced *IL6* mRNA expression in amnion, while Adalimumab decreased its expression in both amnion and chorion. The inhibitors did not have efficacy in decidua parietalis. Data are mean ± SEM, *p < 0.05 (Mann–Whitney U-test).

**Figure 6:**
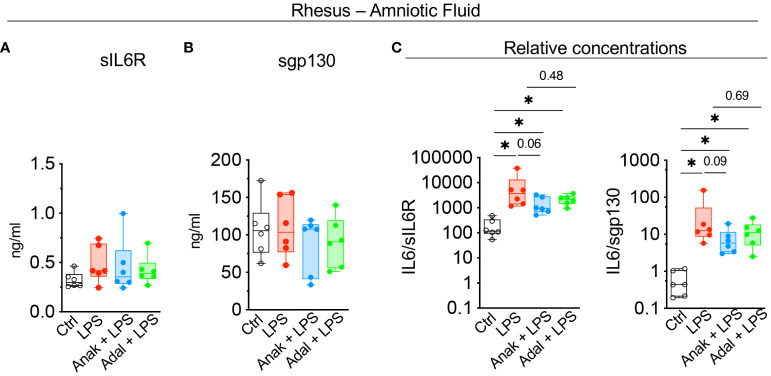
LPS-exposure significantly increased IL6/sIL6R and IL6/sgp130 ratios in the amniotic fluid. Cytokine concentrations were measured in amniotic fluid by ELISA. **(A)** Soluble IL6R (sIL6R) and **(B)** soluble gp130 (sgp130) protein levels did not change after LPS exposure. **(C)** Graph shows a significant increase in IL6/sIL6R and IL6/sgp130 ratio after LPS-exposure. Data are mean ± SEM, *p < 0.05 (Mann–Whitney U-test) (Ctrl n=6; LPS n=6; Anak + LPS n=6; Adal + LPS n=6).

Next, we assessed the contribution of amnion, chorion, and decidua parietalis separately as in human subjects. Rhesus IUI induced by LPS increased *IL6* mRNA expression in the amnon, chorion, and decidua parietalis with the highest fold increase in the amnion (**Figure 5**). Both IL1 and TNF inhibition significantly decreased LPS-induced *IL6* expression in the amnion (**Figure 5**). TNF-blockade but not IL1-blockade decreased significantly *IL6* expression in the chorion (**Figure 5**). On the contrary, the inhibitors did not decrease *IL6* mRNA expression in the decidua parietalis (**Figure 5**). To quantify trans-signaling, we measured sIL6R and sgp130 levels in the Rhesus amniotic fluid (AF). We previously reported that LPS-exposure increased significantly IL6 levels in AF and these were decreased by both IL1- and TNF-inhibition (26, 27). However, LPS did not significantly alter soluble IL6 receptor (sIL6R) (**Figure 6A**) and sgp130 protein levels in the AF (**Figure 6B**). The ratios of IL6/sIL6R and IL6/sgp130 increased significantly upon LPS exposure compared to saline controls (**Figure 6C**). IL1-, but not TNF-inhibitor non-significantly decreased the LPS induced IL6/sIL6R (p=0.06) and IL6/sgp130 (p=0.09) ratios (**Figure 6C**).

### Spatial expression of *IL6* in the Rhesus macaque

**Figure 7:**
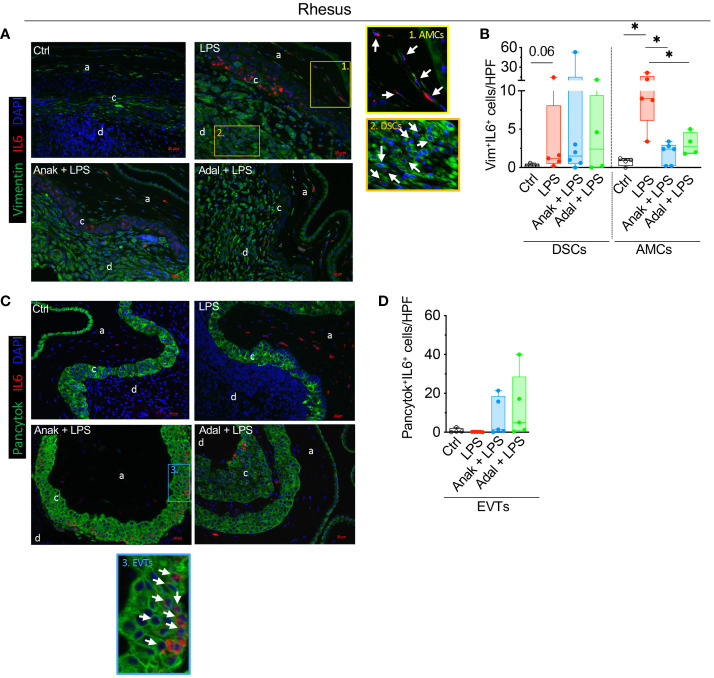
LPS exposure increased significantly the number of rhesus *IL6* ^+^ stroma cells in the decidua parietalis and *IL6* ^+^ amnion mesenchymal cells. Fixed fetal membranes (chorion-amnion-decidua parietalis) paraffin embedded sections were stained by immuno-colocalization. **(A)** Representative images showing *IL6* mRNA identified by RNAscope *in situ* hybridization and Vim colocalization by immunofluorescence. *IL6* is shown in red and Vim in green. White arrows in the magnified yellow inset 1 indicate Vim^+^ *IL6* ^+^ Amnion mesenchymal cells (AMCs), while white arrows in the magnified orange inset 2 indicate Vim^+^IL6^+^ Decidua stroma cells (DSCs) in LPS-exposed animals. **(B)** Quantification of Vim^+^ DCSs and Vim^+^ AMCs expressing *IL6* in amnion, chorion, and decidua. **(C)** Representative images showing *IL6* mRNA identified by RNAscope *in situ* hybridization and Pancytok colocalization by immunofluorescence. *IL6* is shown in red and Pancytok in green. White arrows in the magnified blue inset #3 indicate Pancytok^+^ *IL6* ^+^ EVTs in CAMD section of Anak+LPS animal. subjects. DAPI indicates nuclear staining (blue) in all images. **(D)** Quantification of Pancytok^+^ extra villous trophoblast (EVT) cells expressing *IL6* in amnion, chorion, and decidua. For quantification, an average of 5 randomly selected HPF fields were plotted as the representative value for the animal. Counts were performed in a blinded manner. HPF, high-power field. Data are mean ± SEM, *p < 0.05 (Mann–Whitney U-test) (Ctrl n=4; LPS n=5; Anak+LPS n=5; Adal+LPS n=5). HPF, high-power field; a, amnion; c, chorion, and d, decidua.

LPS-exposure significantly increased the number of *IL6* ^+^Vimentin^+^ amniotic mesenchymal cells (AMCs) (**Figures 7A, B**). Both IL1 and TNF inhibition significantly decreased the numbers of LPS induced AMC *IL6* expression (**Figures 7A, B**). In the decidua, LPS nearly significantly increased the number of *IL6* ^+^Vimentin^+^ decidua stromal cells (DSCs) compared to saline controls (p=0.06; **Figures 7A, B**), but IL-1ra and TNF-blockade did not affect the number of LPS induced *IL6* ^+^DSCs (**Figures 7A, B**). In the chorion, the number of *IL6* ^+^Pan-Cytokeratin^+^ extra villous trophoblasts (EVTs) did not increase upon LPS-exposure (**Figures 7C, D**).

## Discussion

**Figure 8:**
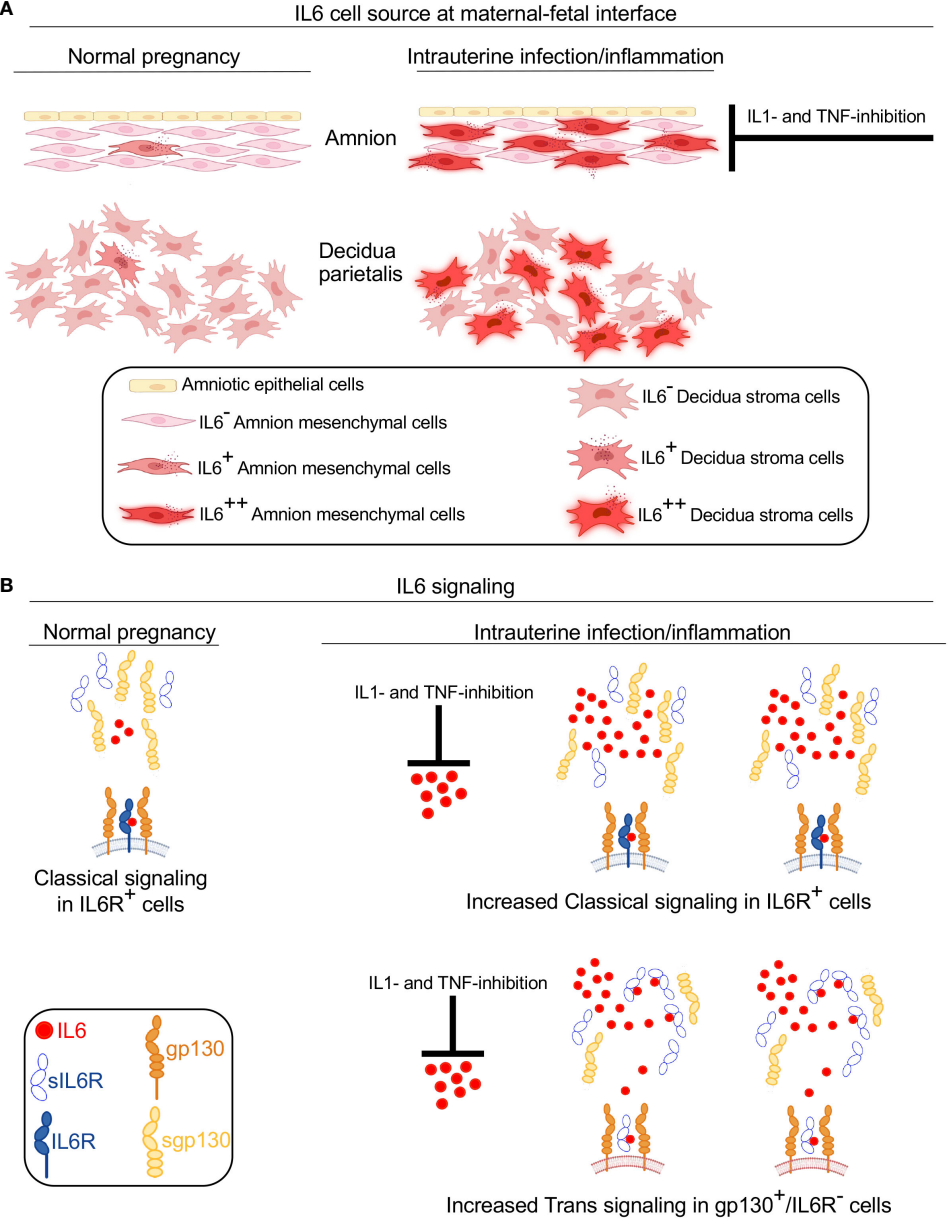
Model for IL6 signaling at the maternal-fetal interface during IUI. A schematic model based on data from Rhesus macaque and human subjects showing source and signaling of IL6 in normal pregnancy and during IUI. **(A)** Under normal conditions, a small amount of IL6 is expressed in the amnion and decidua. IUI significantly increases IL6 expression in activated amnion mesenchymal cells (AMC), decidua stroma cells, and to a small extent in chorion trophoblast cells (not shown). IL6 expression by AMC is inhibited by IL1- and TNF-inhibitors. **(B)** During normal pregnancy IL6 signaling occurs in restricted cells expressing IL6 receptor (IL6R) and gp130 and serves homeostatic function (note both IL6R and gp130 are cell membrane spanning receptors). Soluble IL6R (sIL6R) and soluble gp130 (sgp130) lacking membrane spanning domains are secreted in normal conditions. Due to low levels of IL6 in normal conditions, no significant IL6-sIL6R interactions occur. During IUI, large increase of IL6 expression occurs at the maternal-fetal interface, which is inhibited by IL1- and TNF- inhibitors. Increased IL6 concentration promotes increased number of cells signaling via classical IL6-IL6R-gp130 signaling. sIL6R secretion increases slightly during IUI and sgp130 secretion tends to decrease slightly. Increased IL6 in the inflammatory mileu increases binding to sIL6R. This complex can now bind to membrane spanning gp130 that is expressed ubiquitously. This IL6 trans-signaling increases the number and types of cells responding to IL6 and is pro-inflammatory in nature.

Clinical and experimental evidence strongly demonstrate that IL6 signaling is activated in multiple compartments in maternal and fetal tissues during IUI leading to preterm labor (5–10). While human studies are informative, causal relationship cannot be established since the findings are mainly associative. We therefore paired human studies with a previously validated non-human primate (NHP) model of IUI (26, 27). Main findings of the study are that during IUI the major producers of IL6 are the decidua stroma cells and amnion mesenchymal cells in both preterm Rhesus macaque model of LPS-induced IUI and in preterm human subjects with chorioamnionitis. IL6 trans-signaling, a mechanism known to amplify IL6 signaling (35), is upregulated during IUI. Interesingly, amnion mesenchymal cell but not decidua production of IL6 is inhibited by IL1- or TNF- inhibitors (**Figure 8**). Although 5–12% of term infants are exposed to IUI (36), there is not much known. This study sheds light on IUI in term infants.

Although IL6 can be secreted by a number of different cell-types we found that the main cell types producing IL6 in both human and Rhesus macaque during IUI are the amnion mesenchymal cell (AMC) and decidua stroma cells (DSC). There were subtle differences in cell-type specific IL6 expression during IUI based on gestational age. In term infants, DSC was a more prominent source of IL6, while in the preterm AMC and DSC were both equally involved (**Figure 3**). DSC expression of IL6 during chorioamnionitis was previously reported (37). In the preterm Rhesus macaque, AMC was a more prominent source of IL6 expression during IUI (**Figure 7**). In both the Rhesus macaque and humans, extravillous trophoblast expression of IL6 was detectable but much less prominent compared to AMC and DSC. Amnion expression of IL6 has been reported before but most of the data are from *in vitro* culture studies (38). Interestingly, neutrophils and macrophage expression of IL6 was not detected in both human and NHP subjects during IUI (**Supplementary Figures 2**, **3**). Our results are contradictory to a previous report of chorio-decidua macrophage expression of IL6 during IUI by Wakabayshi et al. (13). The difference is that our study determined *IL6* mRNA expression while Wakabayshi et al. detected IL6 protein by immunohistology. Neutrophil expression of IL6 is controversial, but *in vitro* IL6 expression has been detected after chromatin modification of IL6 locus during inflammation (39). These results emphasize the finding that IL6 (or other cytokine) secretion is context- and stimulus-dependent and therefore *in vivo* demonstration is important rather than assumptions based on *in vitro* studies.

Amnion cell epithelial-to-mesenchymal transition (EMT) and a “mesenchymal state” of amnion was previously shown to be associated with chorioamnionitis, predispose to rupture of membranes, and induce labor (40–43). We recently reported that a subset of AMC get activated during both Rhesus macaque and human IUI. These activated AMC have increased *NF-kB* signaling and express *CD14* and *IL6* in a TNF-dependent manner (30). Consistently, AMC expression of *IL6*, but not DSC *IL6* expression was inhibited by IL1 and TNF blockers (**Figure 7**). In another model of Rhesus macaque IUI induced by live *E.coli*, AMC expression of *IL6* was also reported (14). These data are consistent with TNF and NF-kB signaling dependent expression of AMC in preterm infants exposed to IUI (44). Taken together, data from multiple different Rhesus macaque models and human subjects suggest that AMC secretion of IL6 is a key step in orchestrating host immune response to IUI.

IL6 signaling is relatively unique among cytokines in that a natural soluble IL6 receptor (sIL6R) exists and there is a large increase in tissue levels during inflammation (45). sIL6R is primarily generated via proteolytic cleavage enabled ectodomain sheeding of membrane bound IL6R via ADAM17 (45). Once in circulation, sIL6R binds IL6 and this complex “trans-signals” via membrane bound gp130 heterotrimerizing with IL-6/sIL6R. Since gp130 is ubiquitously expressed, this trans- signaling mechanism not only amplifies IL6 signaling, but can now signals in cells that do not express membrane bound IL6R. This trans-signaling is inhibited by soluble gp130, which is primarily generated by mRNA alternative splicing (45) (**Figure 8**). Recent studies have demonstrated that IL6 preferentially remains in a free form in circulation despite relatively high levels of sIL6R and sgp130 (23) but during inflammation increased IL6 and sIL6R alter the stoichiometry of IL6 complexing with IL6R and sgp130. Clinically, the ratios of IL6/sIL6R and IL6/sgp130 are used as an indicator of IL6 trans-signaling (20–22). We could not quantify IL6 trans-signaling in human subjects in our study due to difficulty in getting amniotic fluid from human subjects, although previous studies demonstrated increased sIL6R in amniotic fluid from women diagnosed with IUI (20). In our Rhesus macaque IUI model, IL6 trans-signaling appeared to be activated as suggested by increased mRNAs for *ADAM17* and *IL6R* in fetal membranes (**Figure 4**), and increased amniotic fluid ratios of IL6/sIL6R and IL6/sgp130 (**Figure 6**). IL1, but not TNF-blockade decreased *ADAM17* expression in fetal membranes exposed to IUI (**Figure 4**), and tended to reduce LPS-induced IL6/sIL6R and IL6/sgp-130 ratios in the amniotic fluid (**Figure 6**).

Intraamniotic injection of IL1β or TNF can increase amniotic fluid and fetal IL6 levels (46, 47), suggesting that both IL1 and TNF are upstream regulators of IL6. We previously published that both IL1 and TNF inhibitors robustly decreased LPS induced amniotic fluid IL6 levels in Rhesus macaque IUI models (26, 27). Taken together, these data lend support to the notion that clinically prescribed IL1 and TNF inhibitors for inflammatory conditions (e.g. rheumatoid arthritis) could be used during pregnancy to reduce IL6 trans-signaling and IUI induced adverse consequences. Although definitive data on safety are not available, IL1 and TNF inhibitors use in pregnancy for pre-existing maternal conditions have been reported to be relatively safe (48, 49). An important consideration with any anti-cytokine therapy is to carefully balance the benefits of inhibiting mal-adaptive immune response during inflammation vs. preserving the beneficial effects of normal immunity orchestrated by the cytokines. Another strategy is to consider anti-inflammatory cytokine therapy such as IL10 for intrauterine inflammation (50, 51).

One important consequence of IUI is preterm birth. We previously reported that LPS-induced IUI does not cause preterm birth in Rhesus macaque (14). There is strong evidence in mice that both normal parturition (inflammation is involved in normal parturition) and inflammation-mediated preterm birth is decreased in the absence of IL6 signaling (11–13, 52). These studies used transgenic (IL6 knock out) or IL6R inhibitors to eliminate/knock down IL6 signaling. IL6 inhition in NHP models of preterm birth has not been reported thus far. Interestingly, intraamniotic injection of IL6 in the NHP does not cause chorioamnionitis and preterm labor (46). A possible explanation for these observations is that IL6 inhibition may be effective in conditions where IL6 trans-signaling is induced, and that injecting IL6 alone may induce classical signaling but may not be able to initiate an inflammatory cascade that results in increased IL6 trans-signaling. Although anecdotal data on IL6 inhibitors (e.g. anti-IL6R antibody Tocilizumab) in pregnant women with inflammatory disorders such as rheumatoid arthritis or severe COVID-19 during pregnancy are reported (53), systematic studies of safety and efficacy have not been reported thus far.

Although IL6 signaling is pro-inflammatory, elegant studies have also demonstrated that in gastro-intestinal mucosal surface, IL6 signaling activating gp130 mediates intestinal epithelial regeneration via YAP and NOTCH signaling (54). Whether fetal membranes repair during chorioamnionitis similar to intestinal epithelium during inflammatory bowel disease is not known.

In summary, our study established close similarities in IL6 signaling during chorioamnionitis/IUI between human subjects (both term and preterm deliveries) and a non-human primate (NHP) model, allowing understanding mechanism of IL6 regulation. During IUI, major sources of IL6 production at the maternal-fetal interface are the amnion-mesenchymal cells and decidua stroma cells and IL6 trans-signaling is induced. The amnion mesenchymal cell *IL6* expression is induced during LPS-induced IUI and inhibited by both IL1 and TNF inhibitors. The experiment adds to our understanding of IL6 biology during intrauterine infection/inflammation.

## Data availability statement

The original contributions presented in the study are included in the article/**Supplementary Material**. Further inquiries can be directed to the corresponding author.

## Ethics statement

Pregnant women provided a written informed consent from 2014 to 2017 under a protocol approved by the Institutional Review Boards (IRBs) of Cincinnati Children’s Hospital and University of Cincinnati (#2013-2243) for use of their placenta samples. The studies were conducted in accordance with the local legislation and institutional requirements. The participants provided their written informed consent to participate in this study. All animal procedures were approved by the IACUC (protocol # 22121) at the University of California Davis Institutional Animal Care and Use Committee and endorsed by the University of California, Los Angeles. The study was conducted in accordance with the local legislation and institutional requirements.

## Author contributions

PP: Data curation, Formal analysis, Investigation, Methodology, Supervision, Validation, Writing – original draft, Writing – review & editing. CR: Formal analysis, Investigation, Methodology, Validation, Writing – original draft, Writing – review & editing. PS: Formal analysis, Investigation, Methodology, Validation, Writing – original draft. MC: Formal analysis, Investigation, Methodology, Writing – original draft. MH: Formal analysis, Methodology, Writing – original draft. LM: Writing – original draft. AJ: Conceptualization, Supervision, Writing – original draft, Writing – review & editing. CC: Conceptualization, Funding acquisition, Resources, Writing – original draft, Writing – review & editing. ED: Resources, Writing – original draft. SK: Conceptualization, Funding acquisition, Investigation, Resources, Supervision, Writing – original draft, Writing – review & editing.

## References

[1] Goldenberg RL Culhane JF Iams JD Romero R. Epidemiology and causes of preterm birth. Lancet. (2008) 371:75–84. doi: 10.1016/S0140-6736(08)60074-4 18177778 PMC7134569

[2] Been JV Lievense S Zimmermann LJ Kramer BW Wolfs TG. Chorioamnionitis as a risk factor for necrotizing enterocolitis: a systematic review and meta-analysis. J Pediatr. (2013) 162:236–42 e2. doi: 10.1016/j.jpeds.2012.07.012 22920508

[3] Venkatesh KK Leviton A Hecht JL Joseph RM Douglass LM Frazier JA. Histologic chorioamnionitis and risk of neurodevelopmental impairment at age 10 years among extremely preterm infants born before 28 weeks of gestation. Am J Obstet Gynecol. (2020) 223(5):745.e1–745.e10. doi: 10.1016/j.ajog.2020.05.001 PMC7609587 32387324

[4] Speer CP. Inflammation and bronchopulmonary dysplasia: a continuing story. Semin Fetal Neonatal Med. (2006) 11:354–62. doi: 10.1016/j.siny.2006.03.004 16702036

[5] Combs CA Gravett M Garite TJ Hickok DE Lapidus J Porreco R. Amniotic fluid infection, inflammation, and colonization in preterm labor with intact membranes. Am J Obstet Gynecol. (2014) 210:125 e1–125 e15. doi: 10.1016/j.ajog.2013.11.032 24274987

[6] Chaemsaithong P Romero R Korzeniewski SJ Martinez-Varea A Dong Z Yoon BH. A rapid interleukin-6 bedside test for the identification of intra-amniotic inflammation in preterm labor with intact membranes. J Matern Fetal Neonatal Med. (2016) 29:349–59. doi: 10.3109/14767058.2015.1006620 PMC4776723 25758618

[7] Jacobsson B Mattsby-Baltzer I Hagberg H. Interleukin-6 and interleukin-8 in cervical and amniotic fluid: relationship to microbial invasion of the chorioamniotic membranes. BJOG. (2005) 112:719–24. doi: 10.1111/j.1471-0528.2005.00536.x 15924526

[8] Romero R Yoon B Kenney J Gomez R Allison A Sehgal P. Amniotic fluid interleukin-6 determinations are of diagnostic and prognostic value in preterm labor. Am J Reprod Immunol. (1993) 30:167–83. doi: 10.1111/j.1600-0897.1993.tb00618.x 8311926

[9] Romero R Miranda J Chaiworapongsa T Korzeniewski SJ Chaemsaithong P Gotsch F. Prevalence and clinical significance of sterile intra-amniotic inflammation in patients with preterm labor and intact membranes. Am J Reprod Immunol. (2014) 72:458–74. doi: 10.1111/aji.12296 PMC4192099 25078709

[10] Romero R Gomez R Ghezzi F Yoon B Mazor M Edwin S. A fetal systemic inflammatory response is followed by the spontaneous onset of preterm parturition. Am J Obstet Gynecol. (1998) 179:186–93. doi: 10.1016/s0002-9378(98)70271-6 9704786

[11] Robertson SA Christiaens I Dorian CL Zaragoza DB Care AS Banks AM. Interleukin-6 is an essential determinant of on-time parturition in the mouse. Endocrinology. (2010) 151:3996–4006. doi: 10.1210/en.2010-0063 20610570

[12] Cappelletti M Presicce P Lawson MJ Chaturvedi V Stankiewicz TE Vanoni S. Type I interferons regulate susceptibility to inflammation-induced preterm birth. JCI Insight. (2017) 2:e91288. doi: 10.1172/jci.insight.91288 28289719 PMC5333966

[13] Wakabayashi A Sawada K Nakayama M Toda A Kimoto A Mabuchi S. Targeting interleukin-6 receptor inhibits preterm delivery induced by inflammation. Mol Hum Reprod. (2013) 19:718–26. doi: 10.1093/molehr/gat057 23969038

[14] Cappelletti M Presicce P Feiyang M Senthamaraikannan P Miller LA Pellegrini M. The induction of preterm labor in rhesus macaques is determined by the strength of immune response to intrauterine infection. PloS Biol. (2021) 19:e3001385. doi: 10.1371/journal.pbio.3001385 34495952 PMC8452070

[15] Calabrese LH Rose-John S. IL-6 biology: implications for clinical targeting in rheumatic disease. Nat Rev Rheumatol. (2014) 10:720–7. doi: 10.1038/nrrheum.2014.127 25136784

[16] Vilotic A Nacka-Aleksic M Pirkovic A Bojic-Trbojevic Z Dekanski D Jovanovic Krivokuca M. IL-6 and IL-8: an overview of their roles in healthy and pathological pregnancies. Int J Mol Sci. (2022) 23(23):14574. doi: 10.3390/ijms232314574 36498901 PMC9738067

[17] Scheller J Chalaris A Schmidt-Arras D Rose-John S. The pro- and anti-inflammatory properties of the cytokine interleukin-6. Biochim Biophys Acta. (2011) 1813:878–88. doi: 10.1016/j.bbamcr.2011.01.034 21296109

[18] Rose-John S Jenkins BJ Garbers C Moll JM Scheller J. Targeting IL-6 trans-signalling: past, present and future prospects. Nat Rev Immunol. (2023) 23(10):666–81. doi: 10.1038/s41577-023-00856-y PMC10108826 37069261

[19] Jostock T Mullberg J Ozbek S Atreya R Blinn G Voltz N. Soluble gp130 is the natural inhibitor of soluble interleukin-6 receptor transsignaling responses. Eur J Biochem. (2001) 268:160–7. doi: 10.1046/j.1432-1327.2001.01867.x 11121117

[20] Lee SY Buhimschi IA Dulay AT Ali UA Zhao G Abdel-Razeq SS. IL-6 trans-signaling system in intra-amniotic inflammation, preterm birth, and preterm premature rupture of the membranes. J Immunol. (2011) 186:3226–36. doi: 10.4049/jimmunol.1003587 PMC3800180 21282511

[21] Rabe B Chalaris A May U Waetzig GH Seegert D Williams AS. Transgenic blockade of interleukin 6 transsignaling abrogates inflammation. Blood. (2008) 111:1021–8. doi: 10.1182/blood-2007-07-102137 17989316

[22] von Bismarck P Claass A Schickor C Krause MF Rose-John S. Altered pulmonary interleukin-6 signaling in preterm infants developing bronchopulmonary dysplasia. Exp Lung Res. (2008) 34:694–706. doi: 10.1080/01902140802389693 19085566

[23] Baran P Hansen S Waetzig GH Akbarzadeh M Lamertz L Huber HJ. The balance of interleukin (IL)-6, IL-6.soluble IL-6 receptor (sIL-6R), and IL-6.sIL-6R.sgp130 complexes allows simultaneous classic and trans-signaling. J Biol Chem. (2018) 293:6762–75. doi: 10.1074/jbc.RA117.001163 PMC5936821 29559558

[24] Sims JE Smith DE. The IL-1 family: regulators of immunity. Nat Rev Immunol. (2010) 10:89–102. doi: 10.1038/nri2691 20081871

[25] Ridker PM Rane M. Interleukin-6 signaling and anti-interleukin-6 therapeutics in cardiovascular disease. Circ Res. (2021) 128:1728–46. doi: 10.1161/CIRCRESAHA.121.319077 33998272

[26] Presicce P Park CW Senthamaraikannan P Bhattacharyya S Jackson C Kong F. IL-1 signaling mediates intrauterine inflammation and chorio-decidua neutrophil recruitment and activation. JCI Insight. (2018) 3(6):e98306. doi: 10.1172/jci.insight.98306 29563340 PMC5926925

[27] Presicce P Cappelletti M Senthamaraikannan P Ma F Morselli M Jackson CM. TNF-signaling modulates neutrophil-mediated immunity at the feto-maternal interface during LPS-induced intrauterine inflammation. Front Immunol. (2020) 11:558. doi: 10.3389/fimmu.2020.00558 32308656 PMC7145904

[28] Bukowski R Sadovsky Y Goodarzi H Zhang H Biggio JR Varner M. Onset of human preterm and term birth is related to unique inflammatory transcriptome profiles at the maternal fetal interface. PeerJ. (2017) 5:e3685. doi: 10.7717/peerj.3685 28879060 PMC5582610

[29] Redline RW Faye-Petersen O Heller D Qureshi F Savell V Vogler C. Amniotic infection syndrome: nosology and reproducibility of placental reaction patterns. Pediatr Dev Pathol. (2003) 6:435–48. doi: 10.1007/s10024-003-7070-y 14708737

[30] Presicce P Cappelletti M Morselli M Ma F Senthamaraikannan P Protti G. Amnion responses to intrauterine inflammation and effects of inhibition of TNF signaling in preterm Rhesus macaque. iScience. (2023) 26:108118. doi: 10.1016/j.isci.2023.108118 37953944 PMC10637919

[31] Jackson CM Demmert M Mukherjee S Isaacs T Thompson R Chastain C. A potent myeloid response is rapidly activated in the lungs of premature Rhesus macaques exposed to intra-uterine inflammation. Mucosal Immunol. (2022) 15(4):730–44. doi: 10.1038/s41385-022-00495-x PMC9259482 35314757

[32] Presicce P Senthamaraikannan P Alvarez M Rueda CM Cappelletti M Miller LA. Neutrophil recruitment and activation in decidua with intra-amniotic IL-1beta in the preterm rhesus macaque. Biol Reprod. (2015) 92:56. doi: 10.1095/biolreprod.114.124420 25537373 PMC4342792

[33] Heatley MK Maxwell P Toner PG. The immunophenotype of human decidua and extra-uterine decidual reactions. Histopathology. (1996) 29:437–42. doi: 10.1046/j.1365-2559.1996.d01-516.x 8951488

[34] Daya D Sabet L. The use of cytokeratin as a sensitive and reliable marker for trophoblastic tissue. Am J Clin Pathol. (1991) 95:137–41. doi: 10.1093/ajcp/95.2.137 1704176

[35] Prairie E Cote F Tsakpinoglou M Mina M Quiniou C Leimert K. The determinant role of IL-6 in the establishment of inflammation leading to spontaneous preterm birth. Cytokine Growth Factor Rev. (2021) 59:118–30. doi: 10.1016/j.cytogfr.2020.12.004 33551331

[36] Romero R Pacora P Kusanovic JP Jung E Panaitescu B Maymon E. Clinical chorioamnionitis at term X: microbiology, clinical signs, placental pathology, and neonatal bacteremia - implications for clinical care. J Perinat Med. (2021) 49:275–98. doi: 10.1515/jpm-2020-0297 PMC8324070 33544519

[37] Lockwood CJ Murk WK Kayisli UA Buchwalder LF Huang SJ Arcuri F. Regulation of interleukin-6 expression in human decidual cells and its potential role in chorioamnionitis. Am J Pathol. (2010) 177:1755–64. doi: 10.2353/ajpath.2010.090781 PMC2947272 20724602

[38] Keelan JA Sato T Mitchell MD. Interleukin (IL)-6 and IL-8 production by human amnion: regulation by cytokines, growth factors, glucocorticoids, phorbol esters, and bacterial lipopolysaccharide. Biol Reprod. (1997) 57:1438–44. doi: 10.1095/biolreprod57.6.1438 9408252

[39] Zimmermann M Aguilera FB Castellucci M Rossato M Costa S Lunardi C. Chromatin remodelling and autocrine TNFalpha are required for optimal interleukin-6 expression in activated human neutrophils. Nat Commun. (2015) 6:6061. doi: 10.1038/ncomms7061 25616107

[40] Richardson LS Taylor RN Menon R. Reversible EMT and MET mediate amnion remodeling during pregnancy and labor. Sci Signal. (2020) 13(618):eaay1486. doi: 10.1126/scisignal.aay1486 32047115 PMC7092701

[41] Janzen C Sen S Lei MY Gagliardi de Assumpcao M Challis J Chaudhuri G. The role of epithelial to mesenchymal transition in human amniotic membrane rupture. J Clin Endocrinol Metab. (2017) 102:1261–9. doi: 10.1210/jc.2016-3150 PMC5460731 28388726

[42] Richardson L Menon R. Proliferative, migratory, and transition properties reveal metastate of human amnion cells. Am J Pathol. (2018) 188:2004–15. doi: 10.1016/j.ajpath.2018.05.019 PMC6119821 29981743

[43] Weed S Armistead B Coleman M Liggit HD Johnson B Tsai J. MicroRNA signature of epithelial-mesenchymal transition in group B streptococcal infection of the placental chorioamniotic membranes. J Infect Dis. (2020) 222:1713–22. doi: 10.1093/infdis/jiaa280 PMC7751568 32453818

[44] Toda A Sawada K Fujikawa T Wakabayashi A Nakamura K Sawada I. Targeting Inhibitor of kappaB Kinase beta Prevents Inflammation-Induced Preterm Delivery by Inhibiting IL-6 Production from Amniotic Cells. Am J Pathol. (2016) 186:616–29. doi: 10.1016/j.ajpath.2015.11.004 26796146

[45] Jones SA Scheller J Rose-John S. Therapeutic strategies for the clinical blockade of IL-6/gp130 signaling. J Clin Invest. (2011) 121:3375–83. doi: 10.1172/JCI57158 PMC3163962 21881215

[46] Sadowsky DW Adams KM Gravett MG Witkin SS Novy MJ. Preterm labor is induced by intraamniotic infusions of interleukin-1beta and tumor necrosis factor-alpha but not by interleukin-6 or interleukin-8 in a nonhuman primate model. Am J Obstet Gynecol. (2006) 195:1578–89. doi: 10.1016/j.ajog.2006.06.072 17132473

[47] Kallapur SG Presicce P Senthamaraikannan P Alvarez M Tarantal AF Miller LM. Intra-amniotic IL-1beta induces fetal inflammation in rhesus monkeys and alters the regulatory T cell/IL-17 balance. J Immunol. (2013) 191:1102–9. doi: 10.4049/jimmunol.1300270 PMC3720768 23794628

[48] Brien ME Gaudreault V Hughes K Hayes DJL Heazell AEP Girard S. A systematic review of the safety of blocking the IL-1 system in human pregnancy. J Clin Med. (2021) 11(1):225. doi: 10.3390/jcm11010225 35011965 PMC8745599

[49] De Felice KM Kane S. Safety of anti-TNF agents in pregnancy. J Allergy Clin Immunol. (2021) 148:661–7. doi: 10.1016/j.jaci.2021.07.005 34489011

[50] Thaxton JE Sharma S. Interleukin-10: a multi-faceted agent of pregnancy. Am J Reprod Immunol. (2010) 63:482–91. doi: 10.1111/j.1600-0897.2010.00810.x PMC3628686 20163400

[51] Kammala AK Mosebarger A Radnaa E Rowlinson E Vora N Fortunato SJ. Extracellular Vesicles-mediated recombinant IL-10 protects against ascending infection-associated preterm birth by reducing fetal inflammatory response. Front Immunol. (2023) 14:1196453. doi: 10.3389/fimmu.2023.1196453 37600782 PMC10437065

[52] Farias-Jofre M Romero R Galaz J Xu Y Miller D Garcia-Flores V. Blockade of IL-6R prevents preterm birth and adverse neonatal outcomes. EBioMedicine. (2023) 98:104865. doi: 10.1016/j.ebiom.2023.104865 37944273 PMC10665693

[53] Jorgensen SCJ Lapinsky SE. Tocilizumab for coronavirus disease 2019 in pregnancy and lactation: a narrative review. Clin Microbiol Infect. (2022) 28:51–7. doi: 10.1016/j.cmi.2021.08.016 PMC8381634 34438068

[54] Taniguchi K Wu LW Grivennikov SI de Jong PR Lian I Yu FX. A gp130-Src-YAP module links inflammation to epithelial regeneration. Nature. (2015) 519:57–62. doi: 10.1038/nature14228 25731159 PMC4447318

