# Supplementary material for: IL-1 and TNF mediates IL-6 signaling at the maternal-fetal interface during intrauterine inflammation
Source: Front Immunol. 2024 Jun 4;15:1416162. doi: 10.3389/fimmu.2024.1416162 (PMC11183269; doi:10.3389/fimmu.2024.1416162)
Supplement: Supplementary file 1 [file DataSheet_1.pdf]

Supplementary Figure 1.

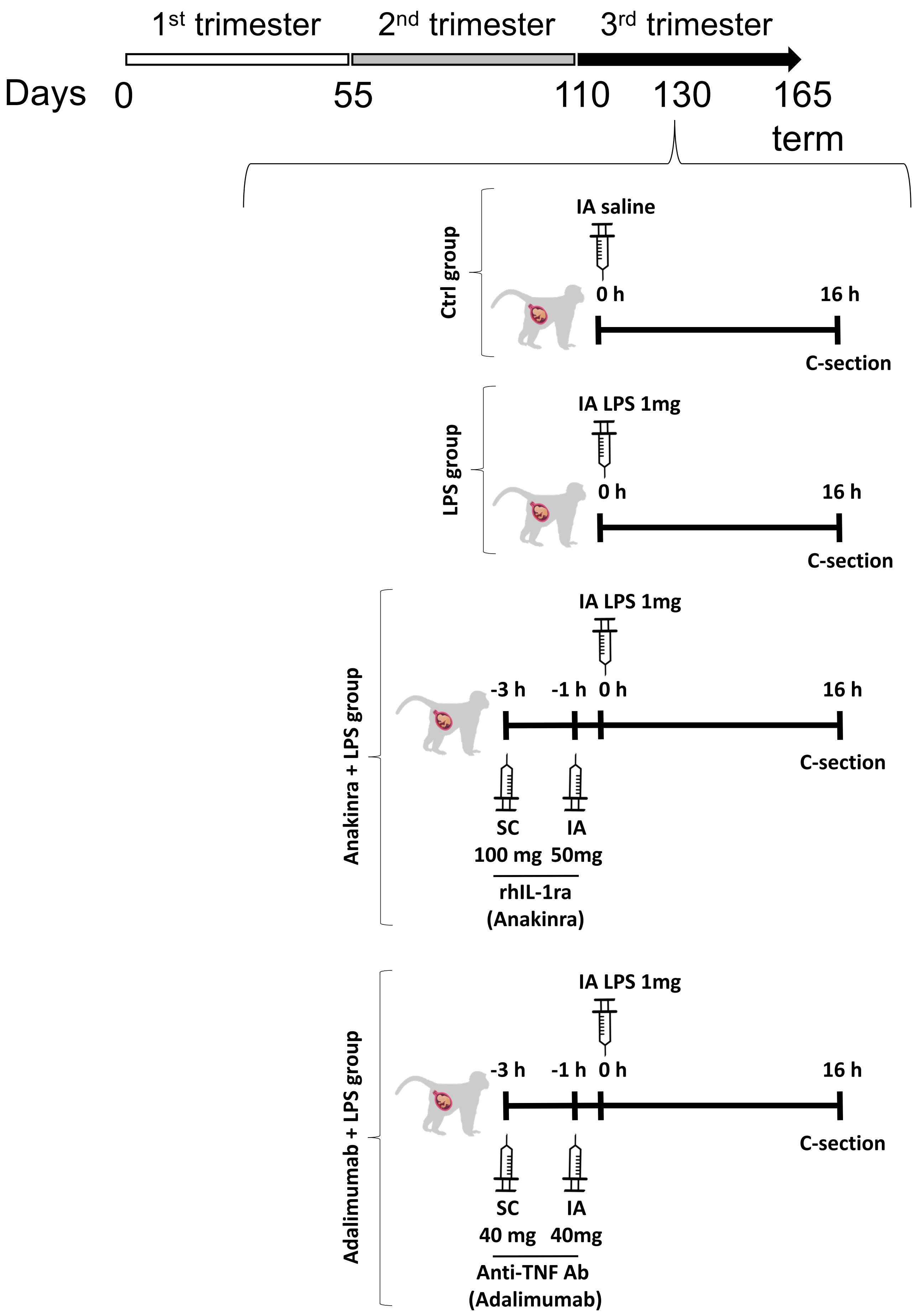

Supplementary Figure 1. Experimental design of the four groups of animals used in the study.

Supplementary Figure 2.

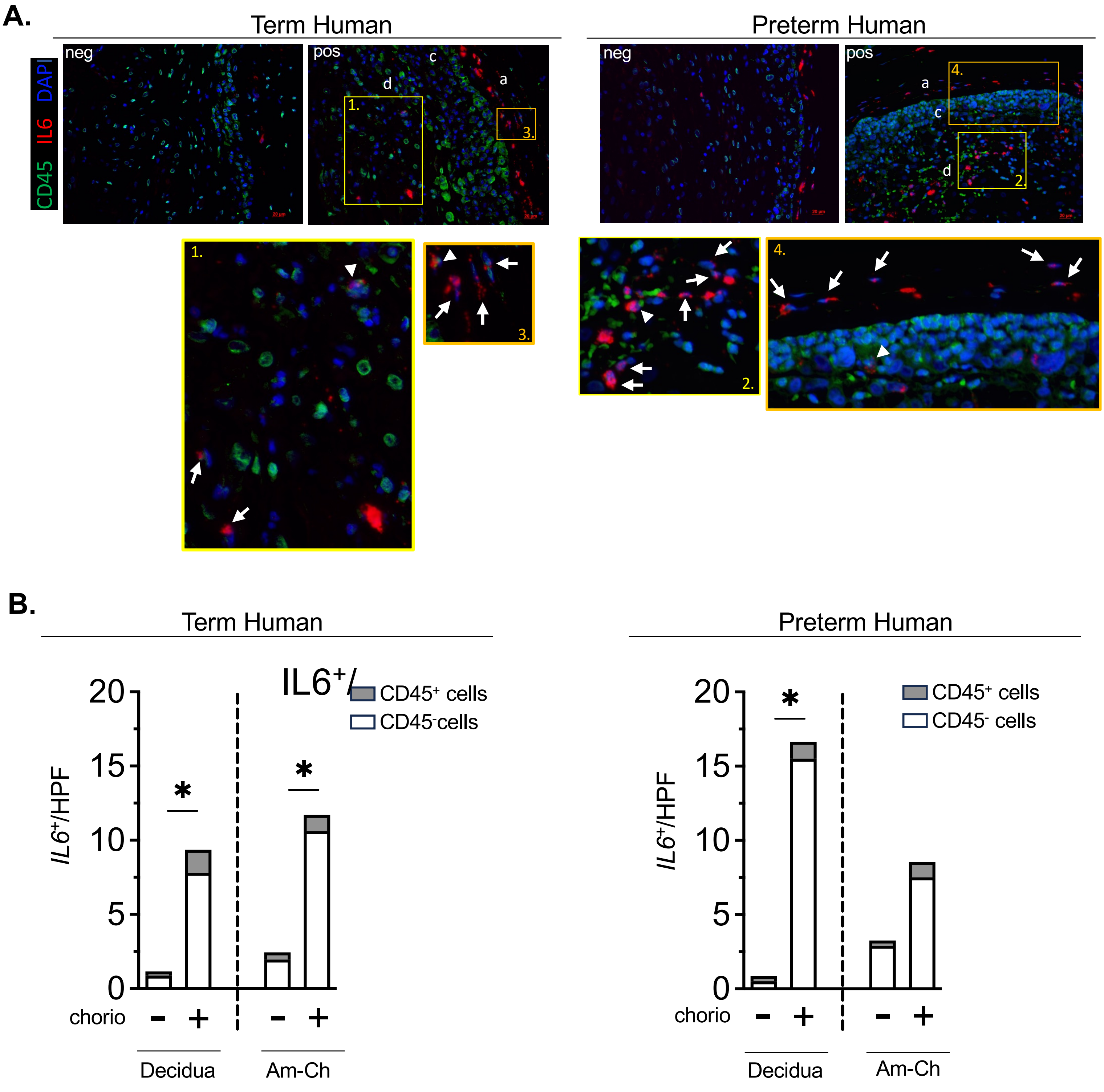

Supplementary Figure 2. $CD45^-$ cells are the major source of *IL6*. Fixed human fetal membranes (chorion-amnion-decidua parietalis) paraffin embedded sections were stained by immuno-colocalization. **(A)** Representative images showing *IL6* mRNA identified by RNAscope *in situ* hybridization and CD45 colocalization by immunofluorescence. *IL6* is shown in red and CD45 in green. Note that the majority of cells expressing *IL6* do not co-express CD45 (indicated by white arrows) and the number of $CD45^+$ immune cells co-expressing *IL6* (indicated by white arrowheads) is very low in magnified yellow insets 1 and 2 (decidua) and orange insets 2 and 4 (amnion-chorion) in both term and preterm samples. **(B)** Quantification of $CD45^-$ non-immune cells (white) and $CD45^+$ immune cells (gray) expressing *IL6* in the decidua and in the Amnion-Chorion (Am-Ch). For quantification, each histogram shows the average of 5 randomly selected HPF fields/sample. Counts were performed in a blinded manner. *$p < 0.05$ Mann–Whitney U-test. Term chorio neg samples n=5; Term chorio neg samples n=4; Preterm chorio neg samples n=5; Preterm chorio neg samples n=4 HPF, high-power field; a = amnion, c = chorion, and d = decidua.

Supplementary Figure 3.

A.

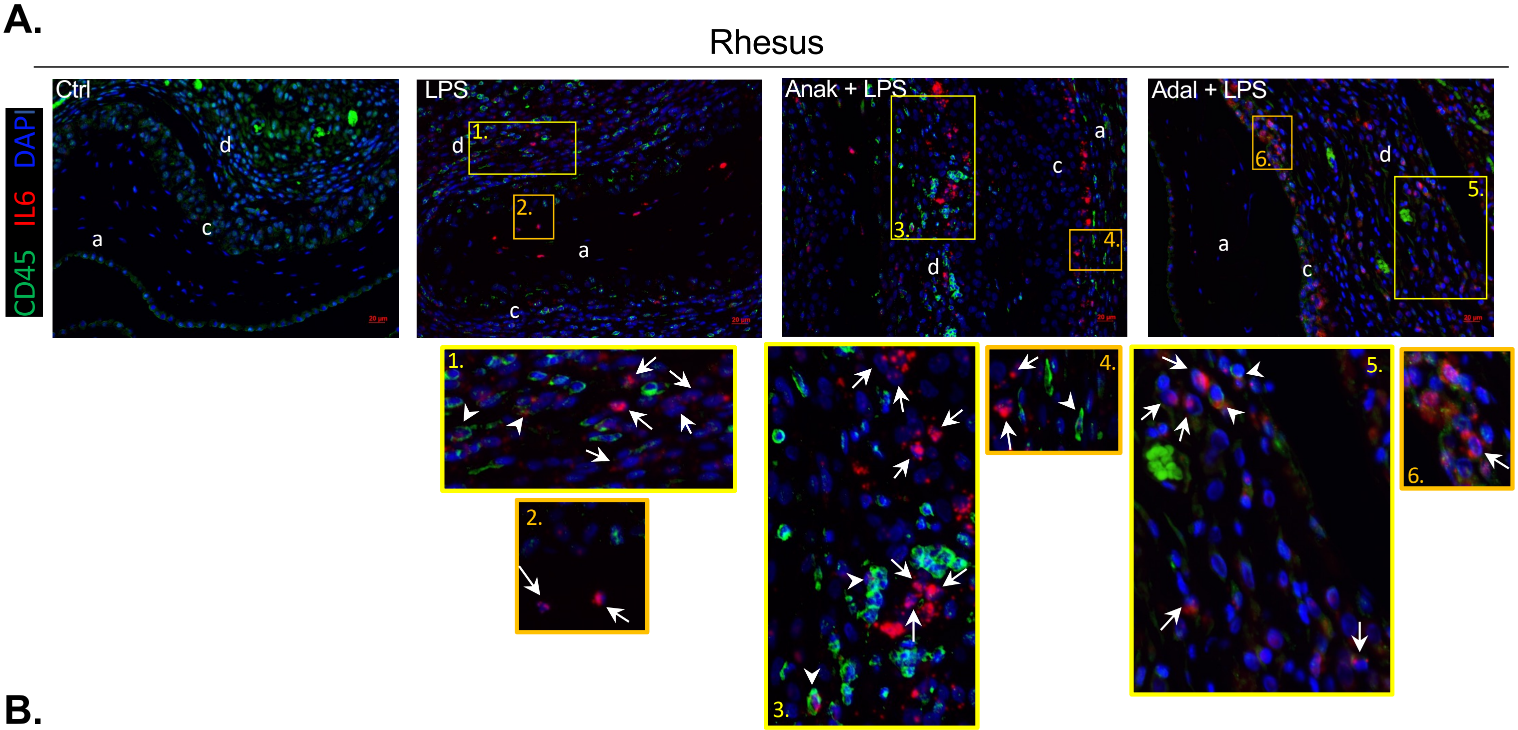

B.

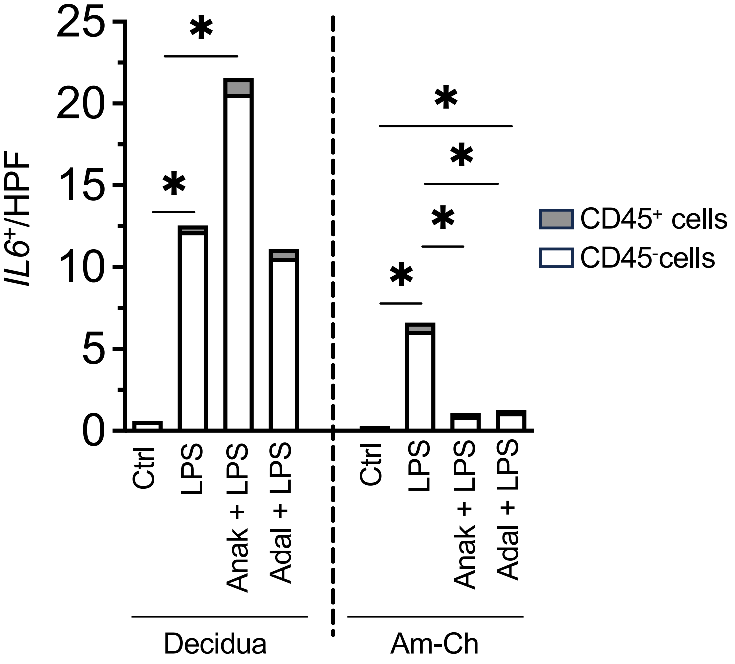

Supplementary Figure 3. Low number of CD45$^+$*IL6*$^+$ immune cells upon LPS-exposure. Fixed fetal membranes (chorion-amnion-decidua parietalis) paraffin embedded sections were stained by immuno-colocalization **(A)** Representative images showing *IL6* mRNA identified by RNAscope *in situ* hybridization and CD45 colocalization by immunofluorescence. *IL6* is shown in red and CD45 in green. Decidua parietalis is magnified in yellow insets #1 (LPS), #3 (Anak + LPS), and #5 (Adal + LPS), while Amnion-Chorion is magnified in yellow insets #2 (LPS), #4 (Anak + LPS), and #6 (Adal + LPS). The majority of CD45$^+$ immune cells do not co-express *IL6*. White arrowheads indicate the few CD45$^+$*IL6*$^+$ immune cells in the decidua, while white arrows indicate CD45$^+$*IL6*$^-$ immune cells. **(B)** Quantification of CD45$^+$immune cells expressing *IL6* in the decidua and in the Amnion-Chorion (Am-Ch). For quantification, an average of 5 randomly selected HPF fields were plotted as the representative value for the animal. Counts were performed in a blinded manner. HPF, high-power field. Data are mean ± SEM, $*p < 0.05$ (Mann–Whitney U-test) (Ctrl n=4; LPS n=5; Anak+LPS n=5; Adal+LPS n=5). HPF, high-power field; a = amnion, c = chorion, and d = decidua.
