# Supplementary material for: IL-1 and TNF mediates IL-6 signaling at the maternal-fetal interface during intrauterine inflammation
Source: Front Immunol. 2024 Jun 4;15:1416162. doi: 10.3389/fimmu.2024.1416162 (PMC11183269; doi:10.3389/fimmu.2024.1416162)
Supplement: Supplementary file 2 [file DataSheet_2.pdf]

Supplementary Table I. Clinical characteristics of pregnant women used in the study.

	Term chorio negative (n=18)	Term chorio positive (n=10)	p value Term chorio neg vs pos	Preterm chorio negative (n=19)	Preterm chorio positive (n=24)	p value Preterm chorio neg vs pos
**Maternal age, year ± SD**	28 ± 7	28 ± 8	0.49#	29 ± 7	28 ± 7	0.82#
**Median GA at delivery, weeks [range]**	39 [37-40]	39 [38-41]	0.42#	32 [26-36]	31 [24-35]	0.23#
**Causes of preterm birth: PTL or preterm-PROM**	0%	0%	n/a	34.2%	77.1%	**<0.0001**‡
**Causes of preterm birth: other indications**	0%	0%	n/a	31.6%	16.7%	0.15‡
**Antenatal steroid use**	0%	0%	n/a	73.7%	87.5%	0.43#
**Antenatal antibiotics use**	0%	37.5%	>0.99‡	21.0%	83.3%	**<0.0001**‡
**Cesarean delivery**	35.3%	50%	0.68‡	57.9%	37.5%	0.22‡
**The presence of labor**	81.2%	90%	>0.99‡	52.6%	95.8%	**0.002**‡
**Spontaneous labor**	63.6%	62.5%	>0.99‡	72.7%	78.3%	>0.99‡
**Neonatal male gender (%)**	50%	31.6%	>0.99‡	31.6%	62.5%	0.06‡
**Mean birth weight, grams ± SD**	3296 ± 361	3337 ± 301	0.55#	1785 ± 536	1699 ± 614	0.95#
**White Caucasian race**	50%	38%	0.67‡	44%	50%	0.76‡
**African American race**	42%	50%	>0.99‡	56%	50%	0.76‡
**Asian race**	8%	12%	>0.99‡	0%	0%	n/a

PTL=preterm labor; PROM=premature rupture of membranes

Other indications: PIH=pregnancy induced hypertension; HTN=hypertension

‡Fisher's exact test

#Student t test

Note: It was not always possible to obtain all the tissues from each human subject. The numbers of subjects for each experiment are shown in the corresponding figure.

Supplementary Table II. Clinical characteristics of animals used in the study.

	Ctrl (Saline) n=27 (IA n=25; IM n=2)	IA LPS n=23	IA/SC Anakinra + IA LPS n=13	IA/SC Adalimumab + IA LPS n=14
**Maternal age, year ± SD**	10 ± 3	8 ± 3	9 ± 2	10 ± 3
**Maternal weight (Kg)**	9.4 ± 1.5	9 ± 2	9.3 ± 1.1	9.9 ± 1.4
**Median gestational age at delivery, day [range]**	131 [129-136]	131 [128-137]	133 [128-135]	132 [124-136]
**Mean birth weight, grams ± SD**	340.1 ± 39.8	316.6 ± 40.9	336.3 ± 36.3	354.7 ± 36.4
**Neonatal male gender (%)**	73%	30%	46%	43%

IA=intraamniotic; IM=intramuscular; SC=subcutaneous

Note: It was not always possible to obtain all the tissues/fluids from each animal. The numbers of animals for each experiment are shown in the corresponding figure.
